# Dual inhibition of EGFR and IL-6-STAT3 signalling by miR-146b: a potential targeted therapy for epithelial ovarian cancer

**DOI:** 10.1080/14756366.2021.1963240

**Published:** 2021-08-08

**Authors:** Meina Yan, Mutian Han, Xinxin Yang, Rong Shen, Hui Wang, Lubin Zhang, Sheng Xia, Peifang Yang, Guanghua Zhai, Qixiang Shao

**Affiliations:** a Department of Laboratory Medicine, The Affiliated Suzhou Hospital of Nanjing Medical University, Suzhou Municipal Hospital, Gusu School, Nanjing Medical University, Suzhou, Jiangsu, P. R. China; b Center of Reproduction and Genetics, Gusu School, The Affiliated Suzhou Hospital of Nanjing Medical University, Suzhou Municipal Hospital, Nanjing Medical University, Suzhou, Jiangsu, P. R. China; c Department of Immunology, School of Medicine, Key Laboratory of Medical Science and Laboratory Medicine, Reproductive Sciences Institute, Jiangsu University, Zhenjiang, Jiangsu, P. R. China; d Department of Gynecology & Obstetrics, Affiliated Hospital of Jiangsu University, Zhenjiang, Jiangsu, P. R. China

**Keywords:** MiR-146b, EGFR, STAT3, epithelial ovarian cancer

## Abstract

Epidermal growth factor receptor (EGFR) signalling and the interleukin-6 (IL-6)/signal transducer and activator of transcription 3 (STAT3) are aberrantly activated in ovarian cancer. However, inhibition of EGFR signalling in ovarian cancer patients resulted in a disappointing clinical benefit. In this study, we found that EGFR could activate IL-6-STAT3 pathway in ovarian cancer cells. However, we also demonstrated that EGFR knockdown could increase STAT3 phosphorylation in HO8910 and OVCAR-3 ovarian cancer cells. Interestingly, we further demonstrated that the non-coding RNA miR-146b could simultaneously block both the EGFR and IL-6-STAT3 pathways. Finally, our data demonstrated that miR-146b overexpression resulted in a greater suppression of cell migration than STAT3 pathway inhibition alone.These results suggest a complex and heterogeneous role of EGFR in ovarian cancer. Combined blockade of EGFR and IL-6-STAT3 pathways by miR-146b might be a strategy for improving the clinical benefit of targeting the EGFR pathway in ovarian cancer patients in the future.

## Introduction

Ovarian cancer (OC) is the second most common gynaecologic cancer and the most lethal gynaecological cancer worldwide. There are approximately 225,500 new cases and an estimated 140,200 ovarian cancer-related deaths each year^1^. Most ovarian tumours are epithelial ovarian carcinomas (EOCs), and approximately 70% of EOC patients are diagnosed at an advanced stage with metastasis^2^. Despite the progress made in ovarian cancer treatments, the 5-year overall survival rate of advanced ovarian cancer is 10–40%^3^. This is primarily because most patients with initially advanced stages later suffer from chemotherapy resistance, and there is a lack of innovative effective treatment strategies. Therefore, there is a pressing need to explore the molecular pathogenesis of OC to search for targeted therapeutic strategies.

It has been widely acknowledged that a number of pathways, including the epidermal growth factor receptor (EGFR) and STAT3 pathways, are aberrantly hyperactivated in human ovarian cancer^4^^,^^5^. Targeted inhibition of EGFR with a small molecule kinase inhibitor has significant clinical benefits in lung cancer with EGFR mutations^6^. EGFR might be an attractive therapeutic target since it is overexpressed in 70% of ovarian cancers^7^. However, the application of several different EGFR inhibitors for ovarian cancer treatment has shown limited benefit when these agents are used as single therapies^7^.

The IL-6-STAT3 pathway participates in a wide variety of tumour biological processes in ovarian and other solid cancers^5^^,^^8^^,^^9^. For instance, high levels of IL-6 promoted the progression of malignant ascites in ovarian cancer^10^. In addition, IL-6 promoted the enrichment of ovarian cancer stem cells after platinum treatment^11^. Furthermore, elevated levels of IL-6 stimulated the hyperactivation of STAT3 signalling, which is often correlated with tumour progression^12^. The IL-6-STAT3 pathway contributes to cell proliferation, invasion, and the resistance to chemotherapeutic drugs in ovarian cancer^13–15^. In addition, high levels of phosphorylated STAT3 (p-STAT3) led to widespread peritoneal metastases and correlated with poor survival in ovarian cancer patients^2^^,^^16^. Recent findings confirmed that EGFR could induce STAT3 phosphorylation^14^^,^^17^^,^^18^. However, EGFR inhibitor treatment similarly resulted in increased STAT3 phosphorylation in human ovarian cancer cells^7^. This observation might explain why EGFR signalling inhibitors have unsatisfactory effects in ovarian cancer patients. Thus, a method for blocking the EGFR and STAT3 pathways in ovarian cancer needs to be developed.

Our previous study confirmed that miR-146b expression was decreased in ovarian cancer tissues, and further investigation demonstrated that miR-146b overexpression inhibited ovarian cancer cell migration and invasion^19^. In this study, we found that increased STAT3 activity after EGFR knockdown could partially explain the unsatisfactory results of anti-EGFR targeted therapy in ovarian cancer patients. Moreover, we found that miR-146b exerts a dual inhibitory effect on the EGFR and IL-6-STAT3 pathways pathway in ovarian cancer cells. These results support the notion that miR-146b overexpression might provide a strategy for improving the clinical benefit of EGFR-targeted treatments in ovarian cancer patients.

## Materials and methods

### Human tissue specimens

All ovarian cancer samples and control samples were collected from the Affiliated Hospital of Jiangsu University (Zhenjiang, Jiangsu, China) and Affiliated People’s Hospital of Jiangsu University (Zhenjiang, Jiangsu, P. R. China). The control samples were obtained from patients who suffered from uterine fibroids, abdominal masses and other diseases that required ovarian removal but who did not have uterine or cervical tumours or other malignant tumours. The samples were quickly snap-frozen in liquid nitrogen and preserved at −80 °C until analysis. Informed consent was obtained from all the recruited subjects. The studies of human tissue samples were performed according to the principles outlined in the relevant national law regarding the protection of biomedical research participants. This study was approved by the Medical Ethics Committee of Jiangsu University (Zhenjiang, Jiangsu, P. R. China).

### Cell culture

The EOC cell lines SKOV3 and HO8910 were kind gifts from Prof. Xiaodong Lu (Department of Histology and Embryology, Jiangsu University, Zhenjiang, Jiangsu, China), and the A2780 and OVCAR-3 cell lines were kind gifts from Prof. Xiaoming Zhou (Medical School, Jiangsu University, P. R. China). Immortalised ovarian epithelial cells (HOSEpic cells) were obtained from Prof. Genbao Shao (Reproductive Sciences Institute, Jiangsu University, P. R. China). The human ovarian cancer cell lines SKOV3, HO8910, and HEK293T were maintained in Dulbecco’s modified Eagle’s medium (DMEM, Gibco, Thermo Fisher Scientific Co., Ltd, Shanghai, P. R. China) supplemented with 10% heated-inactivated foetal bovine serum (FBS), penicillin (100 U/ml), and streptomycin (100 mg/ml). Immortalised ovarian epithelial cells (HOSEpic) and the human ovarian cancer cell lines A2780, and OVCAR-3 were maintained in RPMI-1640 medium (Gibco, Thermo Fisher Scientific (China) Co., Ltd, Shanghai, P. R. China) supplemented with 10% heated-inactivated FBS, penicillin (100 U/ml), and streptomycin (100 mg/ml). All the cells were incubated at 37 °C in a humidified 5% CO_2_ atmosphere.

### RNA isolation and real-time PCR

For ovarian tissue RNA extraction, we first repeatedly added liquid nitrogen to the mortar at least 4–5 times to fully precool the mortar, and then, we placed the tissue samples into the precooled mortar for grinding. Finally, 2 ∼ 3 ml TRIzol reagent was added to each mortar when the liquid nitrogen was volatilised. The weight of the tissue was approximately 50 ∼ 100 mg. For cell RNA extraction, the media were discarded, and the cells were washed once with cold PBS. Then, 1 ml TRIzol reagent was added. Next, total RNA was isolated from tissues and cells using TRIzol reagent according to the manufacturer’s instructions (Takara Biotechnology Co., Ltd., Dalian, P. R. China). Single-stranded cDNA was synthesised using Reverse Transcriptase (Takara Biotechnology Co., Ltd., Dalian, P. R. China). The Primers for IL-6 and EGFR were as follows: IL-6 (163 bp) forward: 5′-CTCAATATTAGAGTCTCAACCCCCA-3′, and reverse: 5′-GAGAAGGCAACTGGACCGAA-3′, EGFR (156 bp) forward: 5′-TAGCAGTCTTATCTAACTATGAT-3′ and reverse: 5′-CACTGCTGACTATGTCCCGC-3′, β-actin (148 bp) forward: 5′-GTTGCGTTACACCCTTTCTTG-3′, and reverse: 5′- CACCTTCACCGTTCCAGTTT-3′. The gene expression levels were evaluated by real-time quantitative PCR with SYBR Premix (Takara Biotechnology Co., Ltd., Dalian, P. R. China). Human β-actin mRNA was analysed in each experimental sample as an internal standard.

### Reagents and antibodies

The Flag-EGFR-plasmid was kindly provided by Prof. Yongchang Chen (Department of Physiology, School of Medicine, Jiangsu University, P. R. China). The antibodies used for Western blotting were as follows: anti-GAPDH (#60004–1-Ig, Proteintech, Wuhan, China), anti-IL-6 (#66146-Ig, Proteintech, Wuhan, China), anti-STAT3 (#4904 CST, Danvers, MA, USA), anti-P-STAT3 (Y705) (#9145, CST, Danvers, MA, USA), anti-P-STAT3 (S727) (#49081, CST, Danvers, MA, USA), anti-EGFR (#4267, CST, Danvers, MA, USA), anti-Flag (F1804, Sigma, USA), HRP-conjugated anti-mouse IgG (#70-GAM007, MultiSciences, China), and horseradish peroxidase (HRP)-conjugated anti-rabbit IgG (#70-GAR007, MultiSciences, China).

### IL-6 concentration detection

We collected the cell supernatants of each group for IL-6 concentration detection. First, a certain volume of a capture microsphere mixture was centrifuged at 200 g for 5 min, the supernatant was discarded, the same volume of microsphere buffer was added, and the mixture was vortexed gently and incubated for 30 min in the dark at room temperature. We labelled one tube with the identification number, then pipetted 25 µL of microsphere, 25 µL of cell supernatant and 25 µL of fluorescence detection reagent, and vortexed the solution gently to mix. Then, we incubated the sample for 2.5 h in the dark at room temperature, added 1–3 ml PBS to each tube, and centrifuged the sample 1500 rpm for 5 min. Finally, we added 100–200 µL PBS to each tube, and the samples were analysed on the flow cytometer.

### Western blotting

Western blotting was performed on total lysates according to the protocol. In brief, total proteins were extracted with 1_×_RIPA lysis buffer with protease inhibitors and phosphatase inhibitors. The protein concentrations were measured with a BCA protein assay kit. Equal amounts of proteins from each lysate were separated by 10 or 12% sodium dodecyl sulphate-polyacrylamide gel electrophoresis (SDS-PAGE) and electronically transferred onto PVDF membranes. The membranes were blocked with 5% BSA for 2 h at room temperature. The membranes were then incubated with primary antibodies overnight at 4 °C with gentle shaking, followed by incubation with HRP-conjugated second antibodies at room temperature for 1 h. Finally, the blots were developed using an enhanced chemo luminescence system (ECL, Merck Millipore, USA).

### Cell migration and invasion assays

Cell migration assays were performed using Corning Transwell insert chambers following the manufacturer’s protocol. Cells suspended in 300-µL serum-free DMEM were placed into the upper chamber of the insert with or without BD Matrigel. Ten percent FBS was used as the chemoattractant. After 24 h of incubation, the cells in the upper chamber were carefully removed and the cells that had migrated through the membrane were stained with crystal violet. Five randomly selected fields were chosen to count the cell numbers, the cells that had migrated through the membrane were counted. All the experiments were performed in triplicate.

### Statistics

All the data are presented as the mean ± SD of three independent experiments. The differences between two groups were analysed using Student’s *t* test. Differences were considered to be statistically significant at *p* < 0.05.

## Results

### EGFR and IL-6-STAT3 signalling predicts a poor prognosis in ovarian cancer

**Figure 1.**
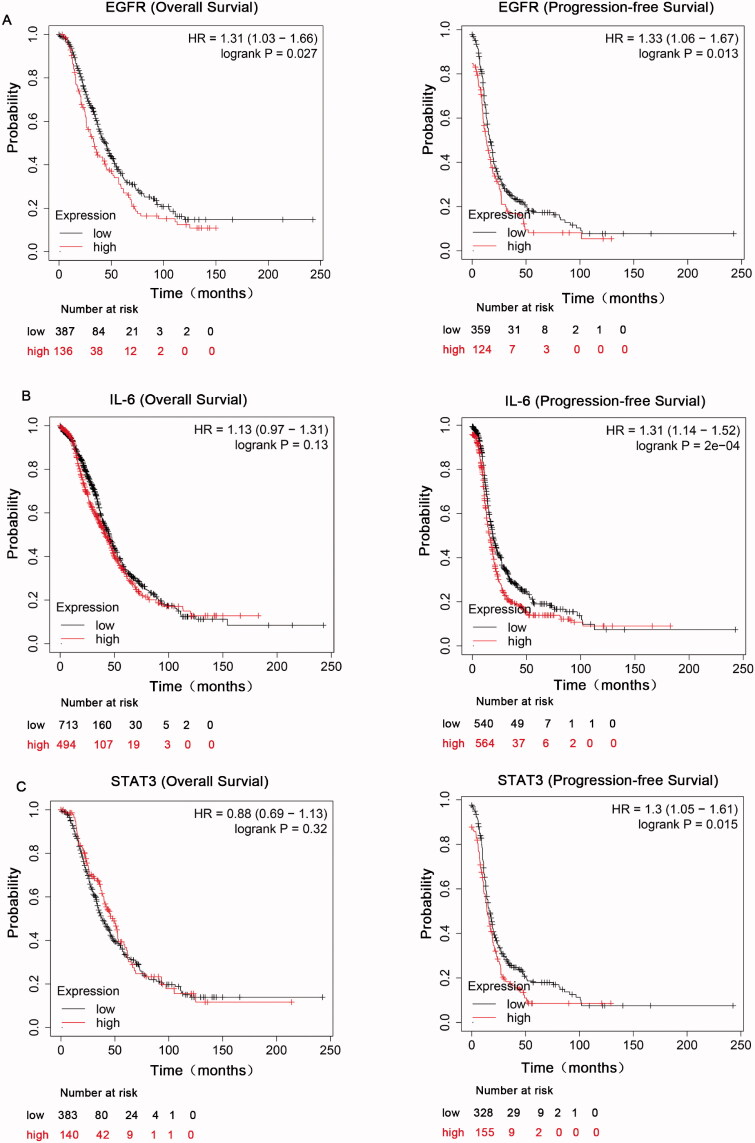
The prognostic value of EGFR and IL-6-STAT3 expression in ovarian cancer. (A) Kaplan–Meier analysis associated with high and low mRNA expression of EGFR in ovarian cancer in http://kmplot.com. (B) The OS and PFS curves were plotted for IL-6 in total ovarian cancer patients in http://kmplot.com. (C) Kaplan–Meier analysis of STAT3 in total ovarian cancer patients in http://kmplot.com.

To explore the roles of EGFR and IL-6-STAT3 in ovarian cancer, we first comprehensively explored the prognostic significance of EGFR and IL-6-STAT3 pathway in patients with ovarian carcinoma using the Kaplan–Meier plotter (KM plotter). We initially evaluated the prognostic value of EGFR in the database. Affymetrix IDs for EGFR: 1565483_at. OS (overall survival) curves and PFS (progression-free survival) curves were plotted for all the ovarian cancer patients. We found that high mRNA expression of EGFR was related to worse OS and PFS in all ovarian cancer patients (Figure 1(A)). We next evaluated the prognostic significance of IL-6 mRNA expression in the database. Affymetrix IDs for IL-6: 205207_at. The results indicated that increased IL-6 mRNA expression had no effect on OS, but was associated with a favourable PFS for all the ovarian cancer patients (Figure 1(B)). Figure 1(C) shows the prognostic value of STAT3 in the database. Affymetrix IDs for STAT3: 225289_at. High STAT3 expression levels had no correlation with OS, but were significantly related to a favourable PFS for all the ovarian cancer patients. Taken together, these findings demonstrated that the EGFR and IL-6-STAT3 pathways have a prognostic value in ovarian cancer.

### Expression of EGFR and IL-6-STAT3 is upregulated in ovarian cancer

**Figure 2.**
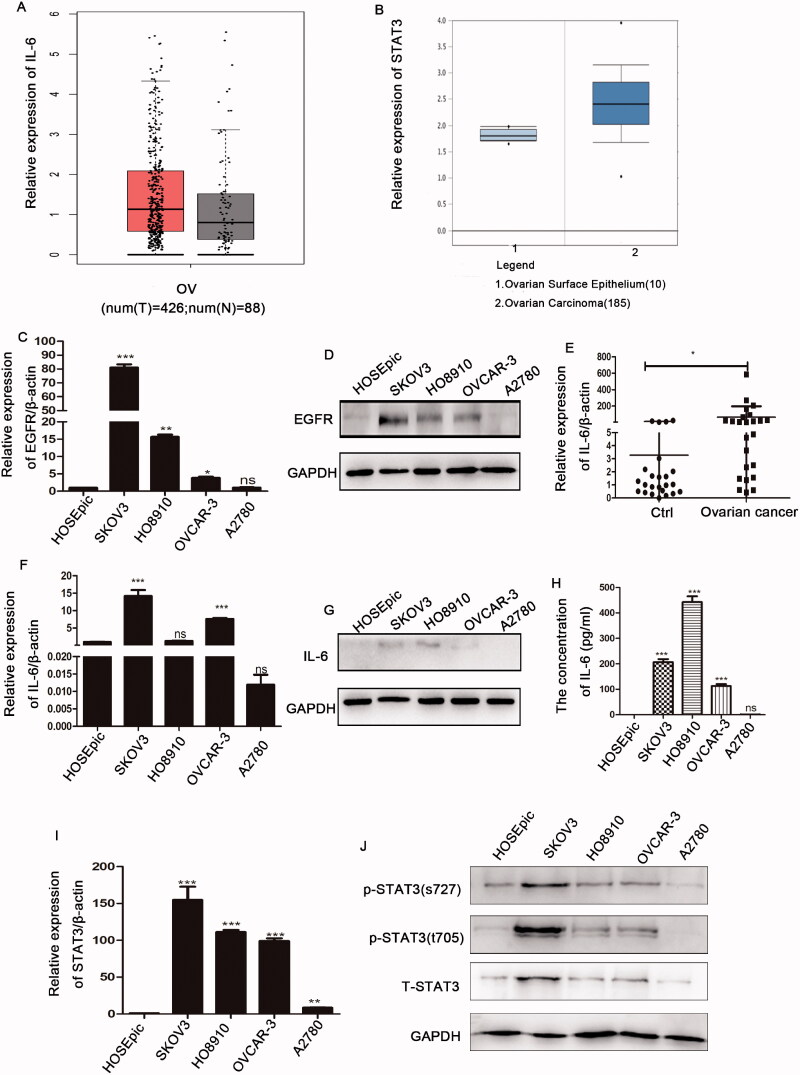
The expression patterns of EGFR and IL-6-STAT3 in ovarian cancer. (A) Analysis of of IL-6 expression in ovarian cancer samples from TCGA database at http://gepia.cancer-pku.cn/. (B) Analysis of STAT3 expression in ovarian cancer tissues from the https://www.oncomine.org database. (C, D) The mRNA and protein levels of EGFR in ovarian cancer cell lines as determined by qPCR and Western blotting. (E) Analyses of mRNA levels of IL-6 in ovarian cancer samples (*n* = 24) and control samples (*n* = 24). (F) The relative mRNA levels of IL-6 in ovarian cancer cell lines. (G) The protein level of IL-6 as determined by Western blotting in ovarian cancer cell lines. (H) The concentration of IL-6 in cell supernatants cell supernatants as determined by flow cytometry in ovarian cancer cell lines. (I) The relative mRNA levels of STAT3 in ovarian cancer cell lines. (J) The relative expression of p-STAT3 (Y705, S727) and STAT3 in ovarian cancer cell lines was examined by Western blotting, and GAPDH was used as the internal control. The data are expressed as the means ± SDs; ns: not significant; *p < 0.05; **p < 0.01; ***p < 0.001.

To further investigate the role of the EGFR and IL-6-STAT3 pathways, we analysed the expression levels of these molecules in ovarian cancer. We first searched tumour-related databases, and found that IL-6 was highly expressed in ovarian cancer tissues compared with normal tissues as reported in the online database http://gepia.cancer-pku.cn/ (Figure 2(A)). By searching https://www.oncomine.org database, we found that expression of STAT3 in ovarian cancer samples was increased (Figure 2(B)). We then measured the expression of EGFR and IL-6-STAT3 in ovarian cancer cell lines. We demonstrated that EGFR expression was increased in almost all ovarian cancer cell lines compared with the normal ovarian epithelial cell line (HOSEpic) (Figure 2(C,D)). Figure 2(E) shows that IL-6 was highly expressed in ovarian cancer tissues compared with normal tissues. In addition, IL-6 was also highly expressed in almost all ovarian cancer cell lines (Figure 2(F–H)). In accordance with IL-6, STAT3 and p-STAT3 expression was also increased in ovarian cancer cell lines (Figure 2(I,J)). Therefore, these data implied that the aberrant upregulation of EGFR and IL-6-STAT3 expression might contribute to the progression of ovarian cancer.

### EGFR activation promotes IL-6-STAT3 pathway activation in epithelial ovarian cancer cells

**Figure 3.**
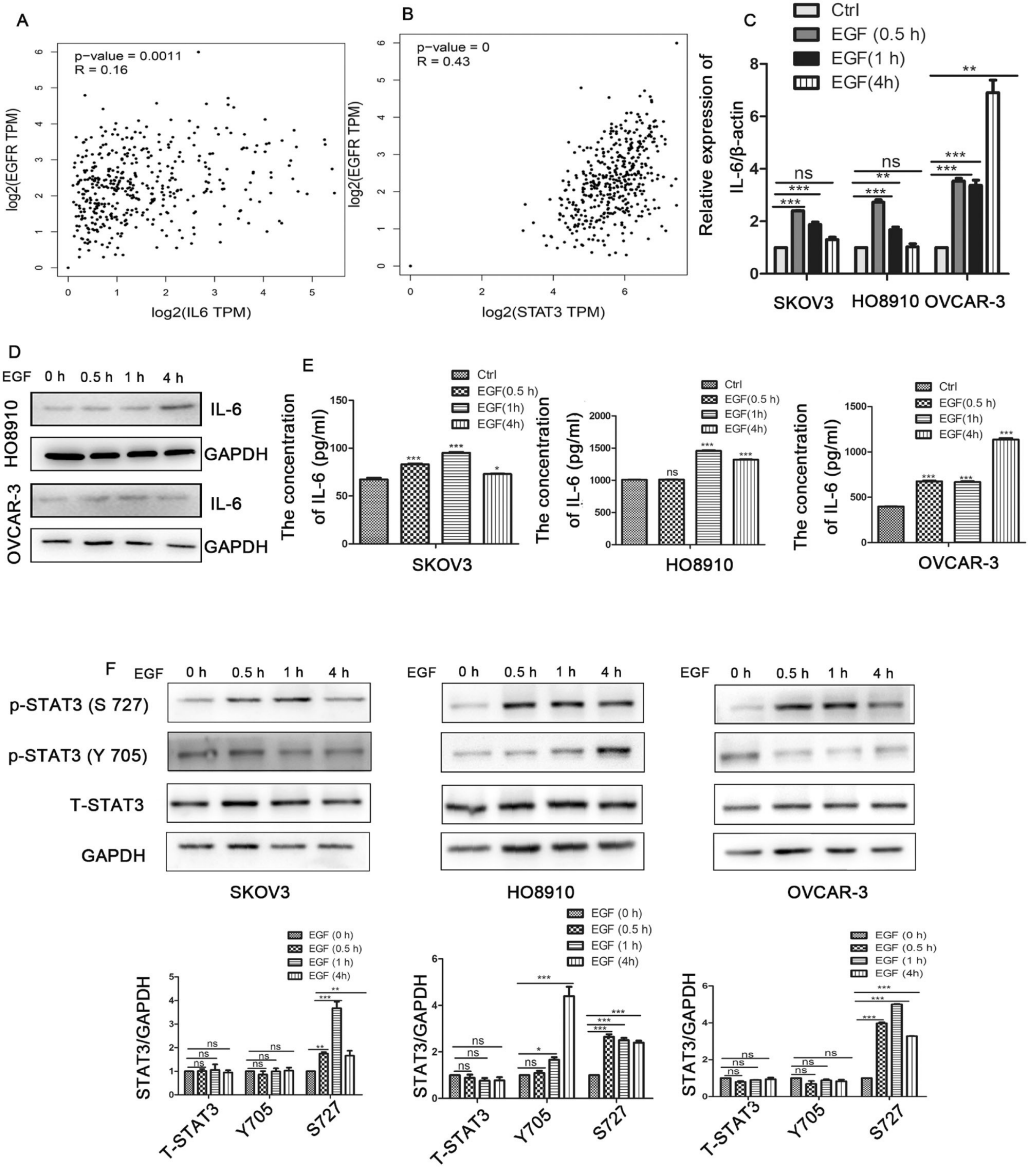
EGFR promotes the IL-6-STAT3 pathway in ovarian cancer. (A) Correlation analysis between EGFR and IL-6 expression in ovarian cancer patients from the online database http://gepia.cancer-pku.cn/. (B) Correlation analysis between EGFR and STAT3 expression in ovarian cancer patients from http://gepia.cancer-pku.cn/. (C,D) The mRNA and protein levels of IL-6 by qPCR and western blot in ovarian cancer cells treated with human recombinant EGF (10 ng/ml) at the indicated times as determined by qPCR and Western blotting. (E) The concentration of IL-6 in cell supernatants as determined by flow cytometry after treatment with EGF. (F) Western blotting analysis of STAT3 expression in ovarian cancer cells treated with human recombinant EGF (10 ng/ml). The data are expressed as the means ± SDs; ns: not significant; *p < 0.05; **p < 0.01; ***p < 0.001.

To further explore the relationship of EGFR and IL-6-STAT3 in ovarian cancer, we analysed the correlation between EGFR and IL-6-STAT3 expression in ovarian cancer via the online database http://gepia.cancer-pku.cn/. As shown in Figure 3(A), the expression of EGFR had no clear correlation with that of IL-6, but it had a positive correlation with the expression of STAT3 in ovarian cancer (Figure 3(B)).Then, we used epidermal growth factor (EGF) stimulation to activate the EGFR pathway. As shown in Figure 3(C–E), EGF stimulation increased both the mRNA and protein levels of IL-6 in ovarian cancer cells. We further confirmed that the level of phosphorylated STAT3 was also increased after treatment with EGF (Figure 3(F)). Therefore, our findings suggested that the IL-6-STAT3 pathway was activated by the EGFR signalling pathway in ovarian cancer.

### MiR-146b blocked the IL-6-STAT3 pathway in ovarian cancer cells

**Figure 4.**
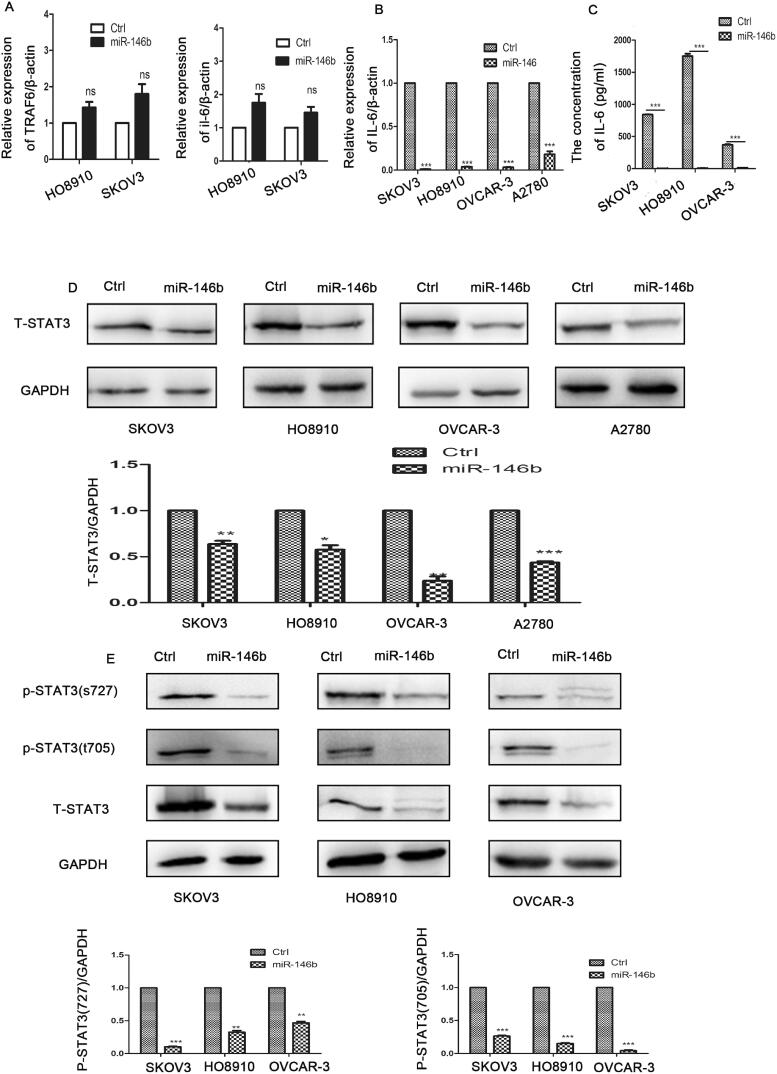
MiR-146b inhibits the IL-6-STAT3 pathway in ovarian cancer. (A) Expression of TRAF6 after miR-146b overexpression in ovarian cancer cell lines as determined by qPCR. (B) The mRNA expression level of IL-6 after miR-146b overexpression. (C) The concentration of IL-6 was detected by flow cytometry after miR-146b overexpression. (D) The effects of miR-146b overexpression on total STAT3 protein expression were analysed using Western blotting and quantified. (E) The effects of miR-146b overexpression on STAT3 phosphorylation were analysed using Western blotting and quantified. The data are expressed as the means ± SDs; ns: not significant; *p < 0.05; **p < 0.01; ***p < 0.001.

Our previous study demonstrated that miR-146b expression was decreased in ovarian cancer tissues compared with normal tissues^19^. A previous study demonstrated that miR-146b inhibited the NF-ᴋB-IL-6-STAT3 pathway by targeting TRAF6 in breast cancer^20^. Accordingly, we investigated whether miR-146b plays the same role in ovarian cancer. We generated a stable subline of ovarian cancer cells that overexpressed miR-146b in our laboratory. We found that miR-146b did not regulate the expression levels of TRAF6 and NF-ᴋB in the ovarian cancer cell lines (Figure 4(A)). However, overexpression of miR-146b markedly reduced the level of IL-6 in the ovarian cancer cell lines (Figure 4(B,C)). Figure 4(D) further indicated that miR-146b overexpression reduced the levels of total STAT3 in the ovarian cancer cell lines. We then examined the level of phosphorylated STAT3 protein and found that tyrosine phosphorylation and serine phosphorylation of STAT3 were both significantly decreased after miR-146b overexpression in the ovarian cancer cell line (Figure 4(E)). These findings established that miR-146b significantly blocked the IL-6-STAT3 pathway in an ovarian cancer cell lines.

### EGFR is a target of miR-146b in ovarian cancer but is not the main regulator of the IL-6-STAT3 pathway

**Figure 5.**
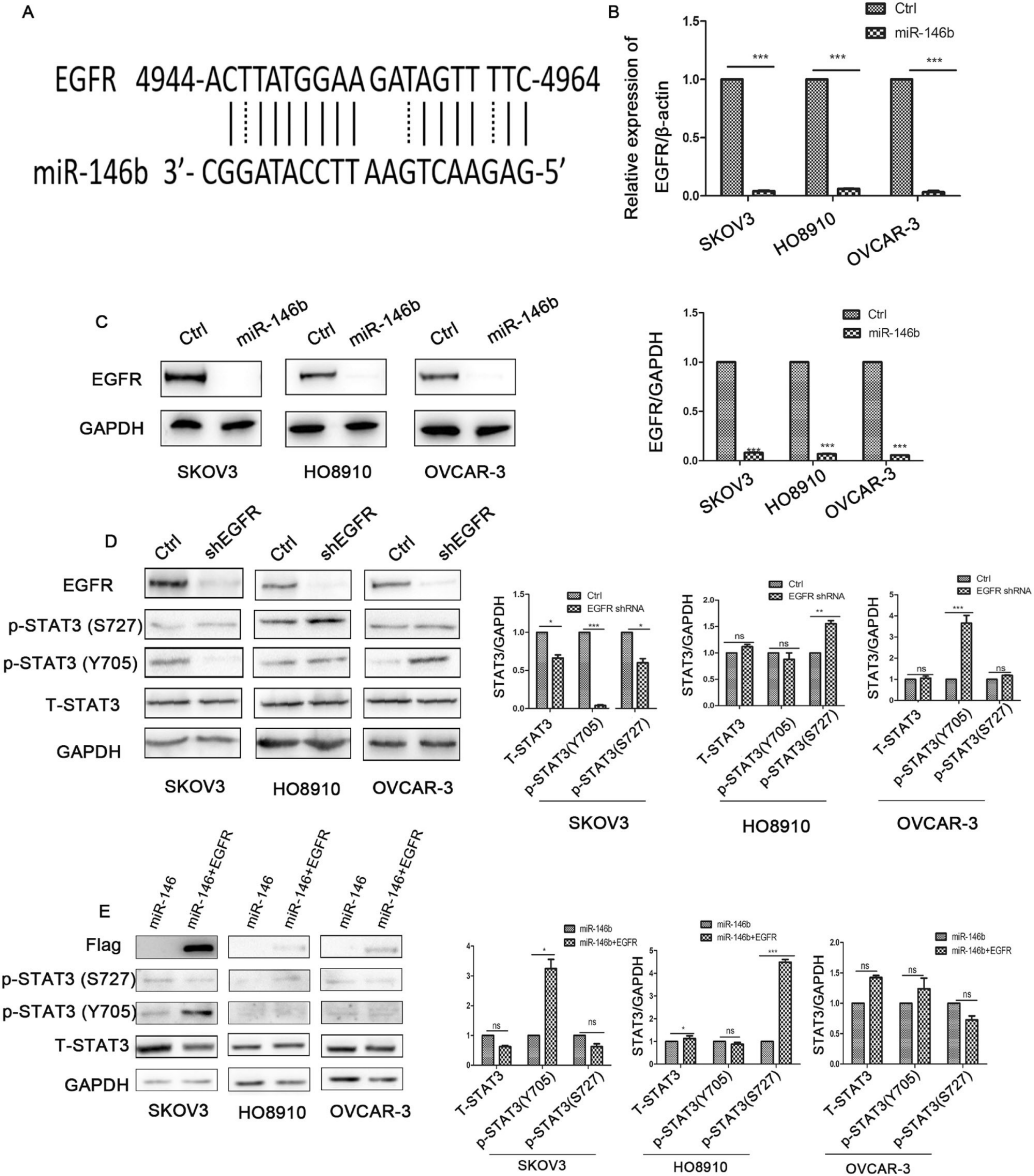
MiR-146b targets EGFR in ovarian cancer. (A) EGFR is a predicted target of miR-146b. (B) The mRNA expression level of EGFR after miR-146b overexpression. (C) The effects of miR-146b on EGFR protein expression were analysed using Western blotting and quantified. (D) After EGFR knockdown by shRNA, the expression of STAT3 was detected by Western blotting. (E) Western blotting analysis of STAT3 of the miR-146b-overexpressing cells that also overexpressed the EGFR ORF. The data are presented as the mean ± SDs of three independent experiments; ns: not significant; *p < 0.05; **p < 0.01; ***p < 0.001.

Our results raised the question, how does miR-146b regulate the IL-6-STAT3 pathway? A previous study demonstrated that EGFR was a validated target of miR-146b^21^. EGFR was also discovered to be a predicted target of miR-146b using the online miRBase target prediction site (Figure 5(A)). Our results further demonstrated that miR-146b significantly decreased the mRNA and protein levels of EGFR in ovarian cancer cells (Figure 5(B,C)). We speculated that miR-146b might target EGFR to regulate the IL-6-STAT3 pathway. We further explored the effect of EGFR knockdown on STAT3 expression. Unexpectedly, the data showed that EGFR downregulation decreased the level of STAT3 in SKOV3 cells but increased phosphorylation of STAT3 in HO8910 and OVCAR-3 cells (Figure 5(D)). Then, we attempted to rescue STAT3 expression by expressing wild-type EGFR without its 3′UTR, and discovered that the expression of tyrosine phosphorylation of STAT3 was partly increased after EGFR overexpression (Figure 5(E)). These data suggested that the regulation of STAT3 by miR-146b is not completely dependent on EGFR. Previous research confirmed that treatment with gefitinib (EGFR inhibitor) led to high STAT3 phosphorylation in ovarian cancer^7^. Thus, we surmise that ovarian cancer cells might use STAT3 pathway activation to overcome EGFR inhibition, which leads to anti-EGFR antibody treatment failure. In addition, our data also demonstrated that ovarian cancer has obvious heterogeneity. The same signalling pathway has different regulatory mechanisms in different cell lines.

### Effect of EGFR-IL-6-STAT3 signalling and miR-146b on ovarian cancer cell migration

**Figure 6.**
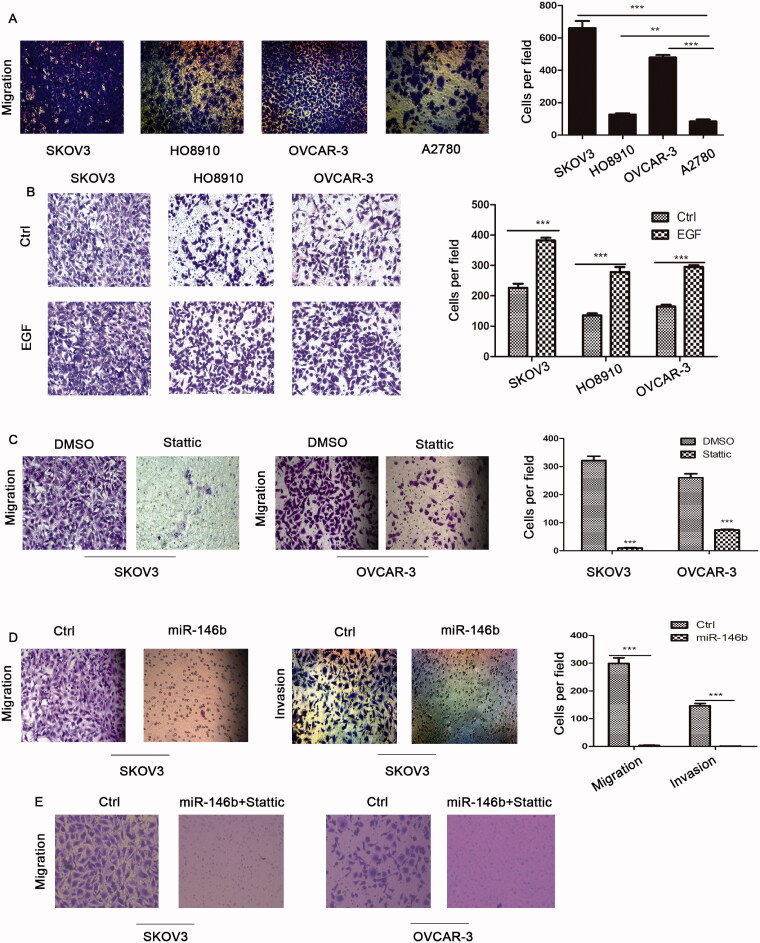
Effect of EGFR-IL-6-STAT3 signalling and miR-146b expression on ovarian cancer cell migration. (A) The migration abilities of EOC cells were determined in a Transwell assay. A total of 1 × 10^5^ cells/well were seeded in the upper chamber of the Transwell insert chambers, and cell migration was assessed according to the manufacturer’s protocol. (B) Effects of EGFR on the migration of ovarian cancer cells. (C) Ovarian cancer cells were treated with 10 μM Stattic for 12 h, and cell migration was assessed using Transwell assays and quantified (*N* = 4 × 10^4^ cells/well).(D) Effects of miR-146b overexpression on the migration and invasion of ovarian cancer cells. The migratory abilities are reflected by the number of cells per microscopic field that had migrated to the underside of the membrane. (E) Effects of miR-146b overexpression and Stattic on the migration of ovarian cancer cells. The data are expressed as the means ± SD; ns: not significant; *p < 0.05; **p < 0.01; ***p < 0.001.

It is well known that cell migration is the key driver of tumour metastasis. Transwell migration assays revealed that EGFR-IL-6-STAT3 pathway activation was linked to increased ovarian cancer cell migration (Figure 6(A)). Moreover, EGF stimulation also promoted ovarian cancer cell migration (Figure 6(B)). Next, we used Stattic, a specific small molecule inhibitor of STAT3, to inhibit STAT3 signalling. Our data showed that ovarian cancer cell migration was decreased after Stattic treatment (Figure 6(C)). We also found that miR-146 significantly inhibited cell migration and invasion in ovarian cancer cells (Figure 6(D)). Finally, we demonstrated that cell migration was completely inhibited in miR146 overexpressing cells treated with Stattic (Figure 6(E)). Altogether, these data suggested that combined targeting both the EGFR and IL-6/STAT3 pathways by miR-146b could result in a greater inhibition of cell migration.

## Discussion

It has been reported that epithelial growth factor receptor (EGFR) plays an active role in a variety of malignancies^22^. Agents that target EGFR signalling signalling, such as gefitinib, erlotinib, and icotinib, have already received approval for the treatment of tumours^23^. EGFR is also a promising potential target for the treatment of ovarian cancer^24^. For example, erlotinib exhibited antiproliferative activity in platinum-resistant ovarian cancer cell lines^25^. However, EGFR inhibitor treatment failed to achieve sufficient clinical benefit ovarian cancer patients when used a single agent or in combination with chemotherapy in ovarian cancer^24^. A study demonstrated that the feedback activation of STAT3 might be the mechanism underlying EGFR inhibitor resistance in ovarian cancer^7^. Similarly, we also found that EGFR knockdown increased STAT3 phosphorylation in HO8910 and OVCAR3 cells. Blockade of the STAT3 pathway might be an effective strategy for increasing the therapeutic efficacy of targeting EGFR in ovarian cancer cells. In this study, we found that high EGFR and IL-6-STAT3 expression predicted a worse survival rate in ovarian cancer patients. We further found that EGFR and IL-6-STAT3 expression was upregulated in ovarian cancer cells. In addition, we demonstrated that EGFR activation could activate the IL-6-STAT3 pathway and result in increased migration of EOC cells. These results suggested that high expression of components of the EGFR-IL-6-STAT3 pathway might be positively correlated with ovarian cancer progression.

MiRNAs are small single-stranded noncoding RNAs that repress the translation or directly promote messenger RNA (mRNA) degradation. MiRNA dysregulation is related to tumour proliferation, apoptosis and invasion. Moreover, miRNA-targeted therapeutics for cancer treatment using miRNA mimics and miRNA antagonists are currently in development^26^^,^^27^. Our previous research showed that miR-146b expression was decreased in ovarian cancer tissues^19^. We further found that miR-146b blocked the secretion of IL-6 and markedly inhibited phosphorylation of STAT3 at Tyr705 and Ser727 in ovarian cancer cells. In addition, our data also indicated that miR-146b targets EGFR in ovarian cancer. A previous study demonstrated that dual inhibition of the EGFR and STAT3 pathways could lead to the simultaneous attenuation of multiple survival pathways in ovarian cancer^7^. Thus, we speculated that miR-146b might provide a potential therapeutic target in ovarian cancer patients.

EGFR could activate a variety of downstream pathways, such as the ERK, AKT and STAT3 pathways, in ovarian cancer cells. Moreover, STAT3 activation was recently suggested to be correlated with the resistance of cells to anti-EGFR therapy^28^^,^^29^. Elevated expression of STAT3 was often observed in the serum and ascites fluid of ovarian cancer patients, and was associated with a poor clinical outcome^16^. To achieve maximum antitumour activity, simultaneous blockade of multiple cancer-promoting pathways might be required. Therefore, miR-146b might improve the antitumour activity of ovarian cancer therapy by blocking both the EGFR and STAT3 pathways. Our data finally demonstrated that miR-146b overexpression appears to be more effective in inhibiting ovarian cancer cell migration than inhibition of the STAT3 pathway alone. Moreover, our data confirmed that miR-146b overexpression combined with Stattic treatment completely suppressed ovarian cancer cell migration.

Taken together, these findings indicated, for the first time, that miR-146b blocks both the EGFR and IL-6-STAT3 pathways in ovarian cancer cells. Our results identify an epigenetic mechanism among miR-146b, EGFR and the IL-6-STAT3 pathway, thus adding to our understanding of how the IL-6-STAT3 pathway is regulated in ovarian cancer. In summary, these findings highlight a vital role of miR-146b in ovarian cancer and might provide insights into its potential use as a strategy to improve the clinical benefit of EGFR-targeted treatment of ovarian cancer.

## Supplementary Material

Supplemental Material
