# Supplementary figures and images for: Dual inhibition of EGFR and IL-6-STAT3 signalling by miR-146b: a potential targeted therapy for epithelial ovarian cancer
Source: J Enzyme Inhib Med Chem. 2021 Aug 8;36(1):1905–15. doi: 10.1080/14756366.2021.1963240 (PMC8354159; doi:10.1080/14756366.2021.1963240)

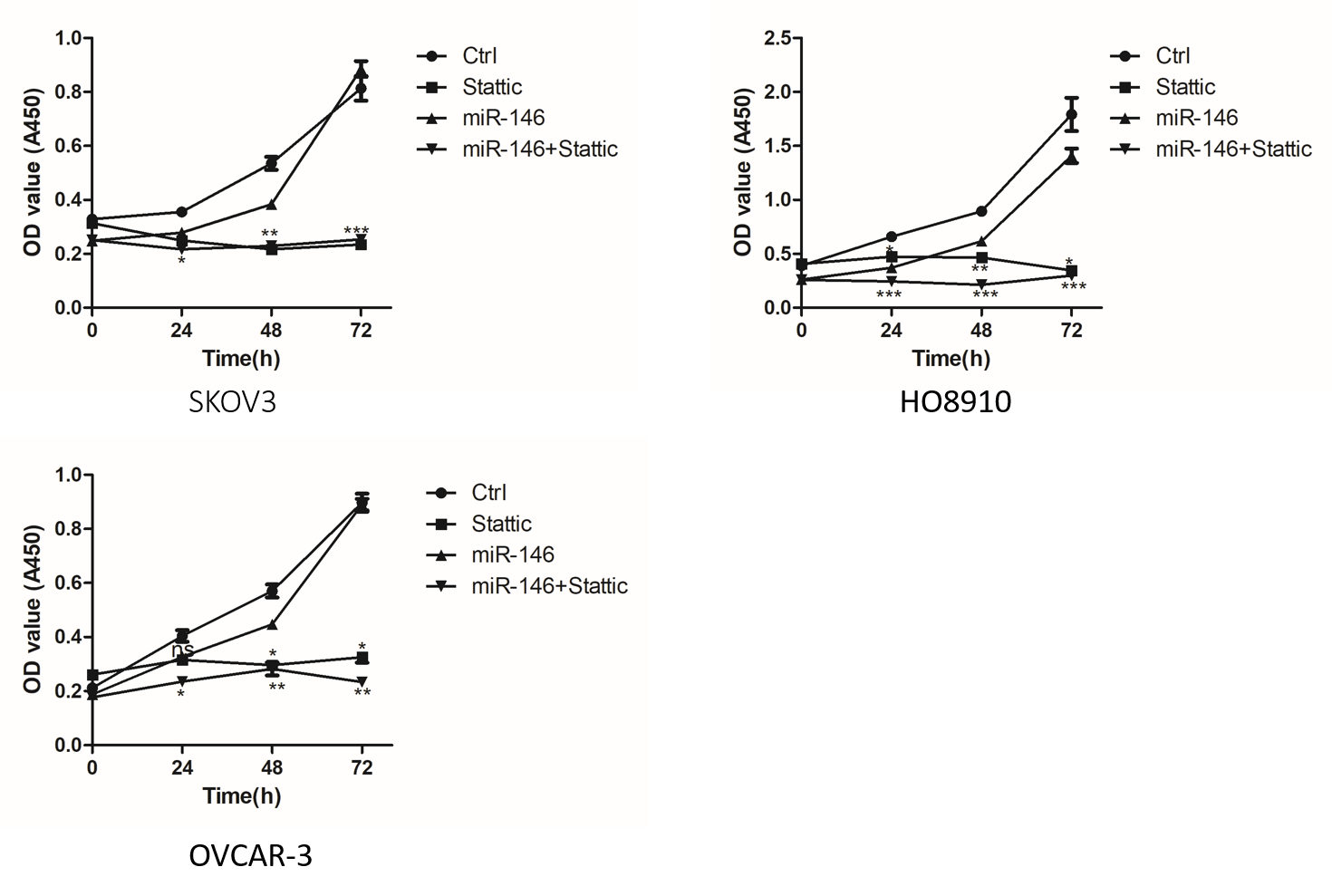

Supplement: Supplemental Material [file IENZ_A_1963240_SM4529.zip › SFig 2.tif]

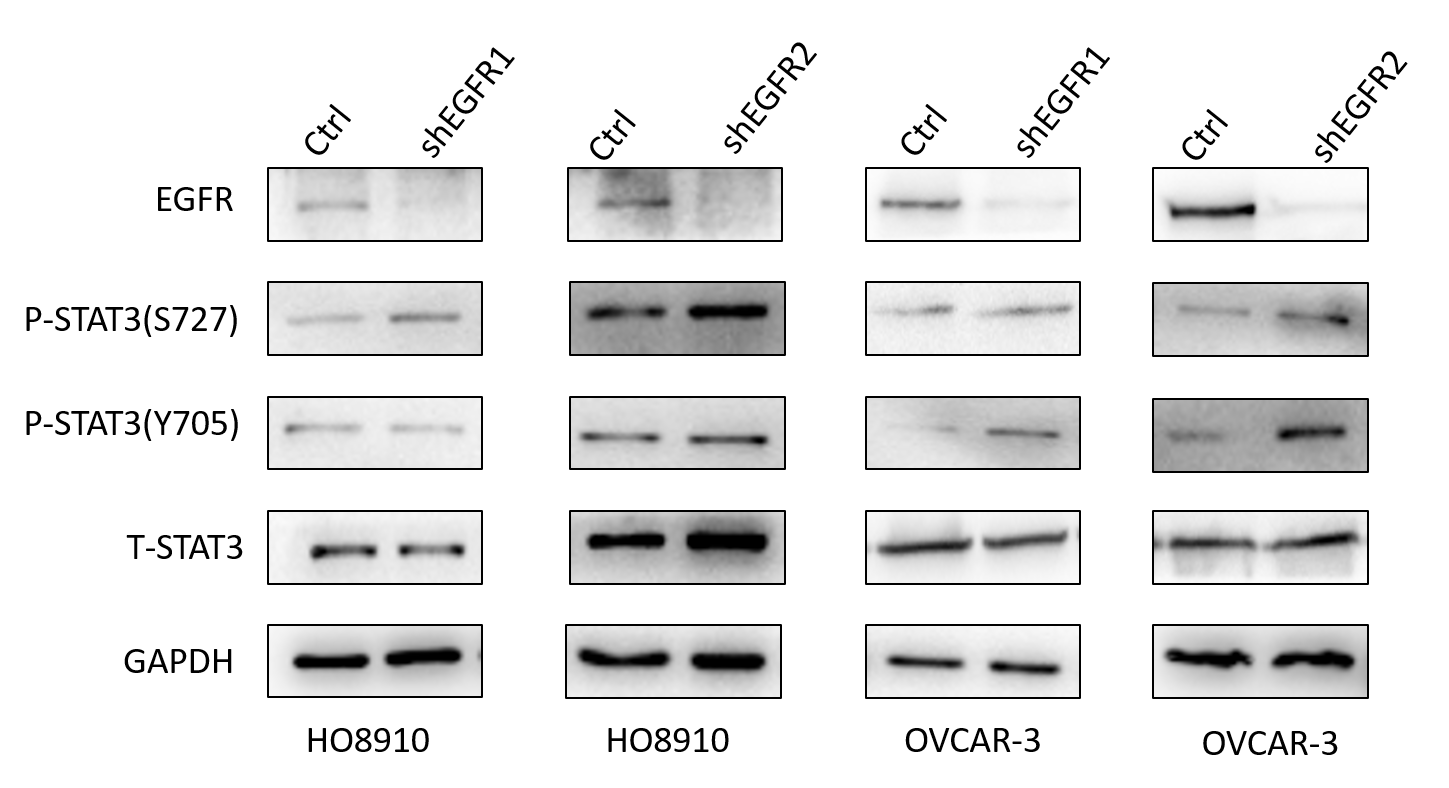

Supplement: Supplemental Material [file IENZ_A_1963240_SM4529.zip › SFig1.tif]
